# Association of active *Helicobacter pylori* infection and anemia in elderly males

**DOI:** 10.1186/s12879-019-3849-y

**Published:** 2019-03-05

**Authors:** Baicun Hou, Meifang Zhang, Miao Liu, Wei Dai, Yunjuan Lin, Yuan Li, Meiliang Gong, Gangshi Wang

**Affiliations:** 1 Department of Gastroenterology, The Second Medical Center, Chinese PLA General Hospital and National Clinical Research Center for Geriatric Diseases, Beijing, 100853 People’s Republic of China; 2 Department of Epidemiology, Institute of Geriatrics, The Second Medical Center, Chinese PLA General Hospital and National Clinical Research Center for Geriatric Diseases, Beijing, 100853 People’s Republic of China; 3 Office of Information Management, The Second Medical Center, Chinese PLA General Hospital and National Clinical Research Center for Geriatric Diseases, Beijing, 100853 People’s Republic of China; 4 Department of Clinical Laboratory, The Second Medical Center, Chinese PLA General Hospital and National Clinical Research Center for Geriatric Diseases, Beijing, 100853 People’s Republic of China

**Keywords:** *Helicobacter pylori*, Anemia, Elderly

## Abstract

**Background:**

The prevalence of Helicobacter pylori *(H. pylori)* infection increases with age. However, the relationship between *H. pylori* infection and anemia in the elderly population remains to be identified. The aim of this study is to explore whether *H. pylori* infection is associated with anemia in a male elderly cohort.

**Methods:**

A cross-sectional study was designed using data collected from asymptomatic male senior citizens (≥ 65 years old) who received an assessment of their health status at the General Hospital of Chinese PLA from January 2015 to December 2015. *H. pylori* infection was confirmed by the ^13^C-urea breath test. Blood samples from the participants were taken to assay for hemoglobin and other erythroid-related indices - serum iron, ferritin, and C-reactive protein (CRP). Anemia was defined as hemoglobin values lower than 120.0 g/L. Charlson Comorbidity Index (CCI) was applied to establish baseline comorbidities.

**Results:**

Data from 646 subjects were analyzed. The mean age of the study cohort was 79.4 ± 8.9 years. The overall prevalence of *H. pylori* infection was 35.3%. The prevalence of anemia in the *H. pylori* positive group was higher than that in the negative group (5.3% vs. 2.2%, *P* = .033). Among the patients who had higher CCI scores (> 2), the prevalence of anemia in the *H. pylori* positive and negative groups were 10.3 and 1.4%, respectively (*P* = .009). Compared to the *H. pylori* negative group, the odds ratio for anemia of the *H. pylori* positive group was 2.53 (P = .033). No correlation between *H. pylori* infection and serum iron and ferritin levels was found. The mean corpuscular volume of the *H. pylori* positive and negative group was 91.17 ± 3.94 fl and 91.17 ± 4.09 fl (mean ± SD), respectively (*P* = .986). The CRP level in the *H. pylori* positive group was higher than that in the *H. pylori* negative group (Median: 0.17 mg/dL vs. 0.10 mg/dL, *P* < .001).

**Conclusion:**

*H. pylori* infection seems to be associated with normocytic and normochromic anemia in elderly males, especially in those with more comorbidities. Further clinical studies are needed to verify the association.

## Background

Epidemiological studies have revealed a correlation between *Helicobacter pylori* (*H. pylori*) infection and some non-gastrointestinal diseases, especially those characterized by persistent and low-grade systemic inflammation [1–3]. Idiopathic iron deficiency anemia (IDA) is a well-recognized extragastric manifestation of *H. pylori* infection and has already been fully accepted and included in the current guidelines for these conditions [1, 2, 4, 5]. After the first report of a young adult whose long-standing IDA was reversed after *H. pylori* eradication [6], some additional trials indicated that *H. pylori* infection is associated with an increased likelihood of depleted iron storage, and *H. pylori* eradication therapy might be beneficial, in terms of increasing ferritin levels [7–10]. Reported data have supported the effectiveness of *H. pylori* treatment in patients with moderate to severe anemia when compared to those with mild anemia [11]. However, it should be noted that the most convincing data for the association between *H. pylori* infection and IDA come from meta-analyses. Meanwhile, there are also reports of conflicting findings on the relationship between *H. pylori* and iron storage [12–15]. Shak JR et al. even found that *H. pylori* sero-positivity was negatively associated with anemia [16].

It has recently been recommended that *H. pylori* infection be tested and treated in persons with unexplained IDA [5, 17]. The consensus was reached based on studies in which most of the subjects were children, adolescents and middle-age adults. There is no guideline or consensus on *H. pylori* management in the elderly population until now. Few data on the effects of *H. pylori* infection on anemia and iron storage in the elderly population were presented. Kaffes et al. observed an association of *H. pylori* infection with significantly lower ferritin levels only in elderly female aspirin users, which indicated that *H. pylori* infection may play a role in iron storage in this population [18]. A small survey performed in asymptomatic elderly subjects found that nutritional indices, including hemoglobin, iron, ferritin and transferrin, were not influenced by the presence of anti-HP antibodies [14]. The relationship between anemia and *H. pylori* infection in the elderly has so far not been established. It is evident that the prevalence of *H. pylori* infection increases with age in developing countries [19–21]. Since medical decision making for the elderly is difficult due to their declining physical and cognitive functions and social support, anemia is associated with an increased mortality risk in persons aged 85 years and older [22], it is meaningful to study if *H. pylori* infection is associated with anemia in this population.

In this study of an asymptomatic elderly cohort presenting active *H. pylori* infection, which was identified with the ^13^C-urea breath test (^13^C-UBT), we examined if they have a higher prevalence of anemia when compared to their counterparts with no evidence of active *H. pylori* infection. Elderly people are often presented with chronic diseases, which are major confounders in longitudinal non-randomized studies. When adjusted for comorbidities, researchers may consider comorbidities individually or through the use of summary measures, such as the Charlson Comorbidity Index (CCI). We applied the CCI scores to adjust the baseline comorbidities, in order to remove the possible confounding factors which may cause anemia in this study. We also assessed the effects of *H. pylori* infection on the level of serum iron, ferritin and C-reactive protein (CRP).

## Methods

### Study subjects

This study is conducted with a cohort of male senior citizens (age ≥ 65 years) who received an assessment of their health status at the Chinese PLA General Hospital from January 2015 to December 2015. All participants received the ^13^C-UBT examination. Exclusion criteria included use of antibiotics, bismuth, or proton pump inhibitor (PPI) and H_2_-receptor antagonist within 4 weeks. On the same day of the ^13^C-UBT, blood samples were collected for complete blood count, and serum iron and ferritin measurements. Serum CRP level was also tested in individuals who have available serum samples. Stool samples were obtained for occult assessment. A detailed clinical history was taken to record any significant acute or chronic illnesses that may cause anemia, such as neoplasia at advanced stages, hematologic disorders, chronic kidney diseases, gastrectomy, malnutrition, acquired immunosuppression, evident gastrointestinal bleeding, and ongoing inflammatory diseases, etc.. In addition, comorbid diseases of each participant were collected and CCI were applied to establish baseline comorbidities. The CCI is based on a number of conditions that are each assigned an integer weight from one to six, with a weight of six representing the most severe morbidity. The summation of the weighted comorbidity scores results in a summary score [23], which can give clinicians and researchers a single number that captures the individual’s health status.

Patients with evident gastrointestinal cancer, hematologic disorders, who received iron supplementation for at least 30 days, received erythropoietin injection, those with overt/occult gastrointestinal bleeding, with malnutrition, a history of gastrectomy, and those with recent hospitalization due to acute illnesses were excluded. This study was approved by the Ethics Committee of the General Hospital of Chinese PLA.

### *H. pylori* status

*H. pylori* status was evaluated by the ^13^C-UBT, which was performed in the morning after fasting for at least 8 h. Breath samples were collected from each subject at baseline and 30 min after drinking 70 mL of room-temperature water containing 75 mg of ^13^C-urea. An additional breath sample was collected 30 min after the ingestion of the tracer. The test was performed with a ^13^C-breath test instrument (Fischer Analysen Instrumente GmbH, Leipzig, Germany). The results were expressed as a surplus of isotopic ratio over the baseline isotopic ratio (delta over baseline [DOB]). A DOB value of > 4 ‰ was considered positive, according to the instructions from the test manufacturer.

### Clinical tests

Blood counts were determined using an automated electronic counter (Sysmex XN3000, Sysmex Corporation, Kobe, Japan). The serum concentration of ferritin was determined by an electrochemiluminescence immunoassay (Cobas e601, Roche Diagnostics Ltd., Switzerland). The serum levels of iron and CRP were also determined by electrochemiluminescence immunoassay (Cobas c501, Roche Diagnostics Ltd., Switzerland). The reference ranges for serum iron and ferritin were 7 - 32 μmol/L and 30–400 ng/mL, respectively. The reference range for mean corpuscular volume (MCV) was 80–100 fl, and that for CRP was 0–0.8 mg/dL. The World Health Organization defines anemia in the adult or elderly populations as hemoglobin concentrations below the level of 120 g/L for non-pregnant women, and 130 g/L for men [24]. The use of this criterion in the elderly population has been questioned because these cutoff levels were derived from statistical distributions in a study in which the participants were healthy and young. We use a hemoglobin concentration of lower than 120 g/L as the cutoff value for anemia in this study, based on both the data described in the literatures and the normal range (110–176 g/L) of the test system we applied. A cutoff value of 130 g/L was used in sensitivity analyses.

### Statistical analysis

Statistical analyses were performed using Statistical Package for SPSS (Windows version 19.0). Continuous variables were expressed as ^−^x ± s for variables of normal distribution and median (interquartile range) for variables of skewness distribution. Categorical variables were expressed as n (%). T test, χ2 test and nonparametric test were used to examine the differences in the corresponding continuous and categorical variables. Multivariable logistic regression was used to calculate the odds ratios (ORs) and 95% confidence intervals (CIs). Two-sided *P* value < .05 was considered as statistically significant.

## Results

### General information and baseline diseases of participants

Results from 646 subjects were available for analysis. The mean age was 79.4 ± 8.9 years, with a range of 65–100 years. Baseline diseases which may cause a decrease in hemoglobin concentration were documented (Table 5 in Appendix). The comorbidities were measured by CCI, with an average CCI score of 1.93 in the 646 subjects. The prevalence of *H. pylori* infection was 35.3%. The subjects were further divided into three subgroups based on their age [65–74 years group (*n* = 239), 75–84 years group (*n* = 162), ≥ 85 years group (*n* = 245)]. As summarized in Table 1, the CCI score and prevalence of comorbidities increased significantly with age (*P* < .001). There is no difference in the levels of hemoglobin, serum iron and ferritin, as well as *H. pylori* prevalence, among the three subgroups (*P >* .05) (Table 1).

**Table 1:** General Characteristics of participants according to different age groups

Characteristics	Age group (years)			*P* value	Total (*n* = 646)
	65~74 years (*n* = 239)	75~84 years (*n* = 162)	≥85 years (n = 245)		
Mean ± SD					
Age (years)	69.48 ± 2.78	79.72 ± 2.97	88.87 ± 3.11	< 0.001	79.40 ± 8.90
Hemoglobin(g/L)	145.91 ± 13.39	145.76 ± 13.09	146.14 ± 13.58	0.959	145.96 ± 13.36
MCV(fl)	91.36 ± 4.47	90.57 ± 3.82	91.39 ± 3.70	0.084	91.18 ± 4.04
Serum iron (μmol/L)	20.47 ± 6.49	19.97 ± 5.55	19.25 ± 5.57	0.076	19.88 ± 5.94
CCI score	1.55 ± 1.27	1.95 ± 1.53	2.28 ± 1.68	< 0.001	1.93 ± 1.53
Median (IQR)					
Serum ferritin (ng/mL)	180.50 (110.80–256.50)	165.50 (109.80–272.75)	186.10 (107.30–273.60)	0.903	179.55 (110.08–268.10)
N (%)					
Malignant tumor	22 (9.2)	30 (18.5)	45 (18.4)	0.005	97 (15.0)
Other comorbidities	13 (5.4)	25 (15.4)	65 (26.5)	< 0.001	103 (15.9)
*H. pylori* positive	85 (35.6)	59 (36.4)	84 (34.3)	0.767	228 (35.3)

### *H. pylori* status and hematological parameters

Twenty-one cases with hemoglobin less than 120 g/L were observed in this cohort (Table 6 in Appendix). In general, the prevalence of anemia in the *H. pylori* positive group was higher than that in the negative group (5.3% vs 2.2%, *P* = .033). The hemoglobin concentration was slightly lower in the *H. pylori* positive group than in the negative group, but with no statistical significance (mean ± SD, 144.83 ± 13.77 g/L vs. 146.57 ± 13.11 g/L, *P* = .115). Either age or CCI score alone had no effects on anemia prevalence or hemoglobin levels (Table 2). When using hemoglobin 130 g/L as a cutoff value for anemia, the odds ratio (OR) of *H. pylori* positive on anemia prevalence was 1.27 (95% CI: 0.78–2.07; *P* = .332), compared with the *H. pylori* negative group (Table 7 in Appendix).

**Table 2:** Anemia prevalence and hemoglobin levels according to age, CCI score and *H. pylori* status

Characteristics	Number of cases	Anemia prevalence		Hemoglobin (g/L)	
		n (%)	*P* value	Mean ± SD	*P* value
Age			0.845		0.959
65–74 years	239	9 (3.8)		145.91 ± 13.39	
75–84 years	162	5 (3.1)		145.76 ± 13.09	
≥ 85 years	245	7 (2.9)		146.14 ± 13.58	
CCI score			0.442		0.891
≤ 2	449	13 (2.9)		145.91 ± 13.42	
> 2	197	8 (4.1)		146.06 ± 13.27	
*H. pylori* status			0.033		0.115
positive	228	12 (5.3)		144.83 ± 13.77	
negative	418	9 (2.2)		146.57 ± 13.11	

The relationship between anemia and *H. pylori* status according to CCI scores and age were further analyzed. The difference of anemia prevalence in each age subgroups remained to be around 3%, but was of no statistical significance. The average CCI scores of *H. pylori* positive and negative group was 1.85 and 1.97, respectively (*P* = .643). No relation between CCI scores and anemia was found in this study population. The prevalence of anemia in high (>2) and low (≤ 2) CCI score groups was 4.1 and 2.9%, respectively (*P* = .442). Among the patients with low CCI score (≤ 2), there was no significant difference of the anemia prevalence between the *H. pylori* positive and negative groups (3.5% vs. 2.5%, *P* = .569). However, among the patients who had higher CCI scores (>2), the prevalence of anemia was higher in the *H. pylori* positive group than those in the negative group (10.3% vs.1.4%, *P* = .009) (Table 3).

**Table 3:** Relationship between anemia and *H. pylori* status according to age and CCI score

characteristic		Number of cases	Anemia prevalence n (%)	Difference (95% CI)	*P* value
Age group	65–74 years				
	*H. pylori* positive	85	5 (5.9)	3.3 (−2.3 to 8.9)	0.202
	*H. pylori* negative	154	4 (2.6)		
	75–84 years				
	*H. pylori* positive	59	3 (5.1)	3.1 (−3.1 to 9.4)	0.266
	*H. pylori* negative	103	2 (1.9)		
	≥ 85 years				
	*H. pylori* positive	84	4 (4.8)	2.9 (−2.1 to 7.9)	0.196
	*H. pylori* negative	161	3 (1.9)		
CCI score	≤ 2				
	*H. pylori* positive	170	6 (3.5)	1.0 (−2.3 to 4.4)	0.569
	*H. pylori* negative	279	7 (2.5)		
	> 2				
	*H. pylori* positive	58	6 (10.3)	8.9 (0.8 to 17.0)	0.009
	*H. pylori* negative	139	2 (1.4)		

Table 4 shows the OR of *H. pylori* status on anemia prevalence. Compared with the *H. pylori* negative individuals, the crude OR of *H. pylori* status on anemia prevalence was 2.53 (95% CI: 1.05–6.09; *P* = .033). We also adjusted for age and CCI scores in the logistic regression. The results were similar. In the sensitivity analysis, we calculated the ORs of *H. pylori* status on anemia among those without malignant tumors (*n* = 549), the age–adjusted OR was 2.30 (95% CI: 0.95–5.55).

**Table 4:** ORs and 95% CI of *H. pylori* positive on anemia prevalence

Variable	OR	95% CI	*P* value
Total population (n = 646)			
Crude	2.53	1.05–6.09	0.033
Age and CCI adjusted	2.57	1.06–6.21	0.036
Population without malignant tumor (n = 549)			
Crude	2.29	0.96–5.54	0.065
Age and CCI adjusted	2.42	0.99–5.89	0.051
Population with normal MCV (*n* = 631)			
Crude	2.58	1.02–6.50	0.045
Age and CCI adjusted	2.56	1.02–6.46	0.040

Erythroid-related indices for both the *H. pylori* positive and negative groups were analyzed. Hematocrit (HCT) had a marginal difference between the *H. pylori* positive and negative groups (*P* = .058). The MCV of the *H. pylori* positive and negative group was 91.17 ± 3.94 fl and 91.18 ± 4.09 fl (mean ± SD, *P* = .986), respectively. Other erythroid-related indices showed no significant difference among either *H. pylori* status (*P >* .05) (Table 8 in Appendix).

### Effect of *H. pylori* status on serum iron, ferritin and CRP levels

Similar serum iron levels were found in the *H. pylori* positive and negative groups (mean ± SD, 19.90 ± 6.24 μmol/L vs. 19.87 ± 5.78 μmol/L, *P* = .961). The results were found to be similar for serum ferritin. The median serum ferritin was 174.00 ng/mL (IQR 108.78–267.88) in the *H. pylori* positive group, and 181.65 ng/mL (IQR 110.68–270.60) in the *H. pylori* negative group (*P* = .613).

Among the subjects recruited in this study, CRP data were available in 262 cases, which included 66 *H. pylori* positive and 196 *H. pylori* negative ones. Although all the subjects represent CRP within the normal range, the CRP level in the *H. pylori* positive group (Median: 0.17 mg/dL, IQR: 0.10–0.69) was higher than that in the *H. pylori* negative group (Median: 0.10 mg/dL; IQR: 0.07–0.23) (*P <* .001).

## Discussion

The prevalence of *H. pylori* infection increases with age, with about 50% of the population infected at ages above 60, and around 10% between 18 and 30 [19]. *H. pylori* infections are usually acquired in early childhood in all countries [25]. Therefore, it is reasonable to assume that chronic *H. pylori* infections in the elderly can cause anemia due to predisposition to gastrointestinal mucosal lesions [26].

Among the 646 elderly male subjects, we found a significant association between *H. pylori* infection and anemia. The prevalence of anemia in the positive group was higher than that in the negative group, with a difference of about 3%. This is consistent with the mainstream opinion that anemia is considered as a complication of *H. pylori* infection [9, 27–29]. A strong association between *H. pylori* infection and anemia (OR 2.53, 95% CI 1.05–6.09) was observed in this study. A similar result was reported in a recent study. Xu et al. found that when compared to *H. pylori* negative groups, the OR of the *H. pylori* positive group was 2.01 (95% CI: 0.92–4.40) for mild anemia and 2.41 (95% CI: 0.91–6.34) for moderate-to-severe anemia in male subjects after adjusting for potential confounders [27]. Because of the low prevalence of anemia in this special cohort, no significant difference of average hemoglobin levels between the *H. pylori* positive and negative groups (mean ± SD, 144.83 ± 13.77 g/L vs. 146.57 ± 13.11 g/L, *P* = .115) was found, although the positive group did show a slightly lower hemoglobin level than the negative group.

In elderly subjects, the “accepted” cutoff values for the diagnosis of anemia vary between countries, regions and laboratories [30]. The hemoglobin concentration is lower on average in older people [31]. A significant association between *H. pylori* infection and anemia was found by using hemoglobin concentration of lower than 120 g/L as the cutoff value for anemia in this study. This association was corroborated by a further sensitivity analysis when using the cutoff value of 130 g/L, in which the OR of *H. pylori* positive on anemia prevalence was 1.27. The anemia prevalence (hemoglobin cutoff value of 120 g/L) in this cohort was 3.3%, which was lower than that reported in the literatures. The anemia prevalence of Chinese city population was recently reported as 9.7% (95%CI: 9.4–10.1%), 6.8% (95%CI: 6.4–7.3%) for male and 12.8% (95%CI: 12.2–13.4%) for female, while 11.4% (95%CI: 9.8–11.3%) for male metropolis more than 60 years-old [32]. In this study, 77 cases of anemia could be identified if the cutoff value was set to 130 g/L. This prevalence (11.9%) is similar to that reported in the literature. Furthermore, we excluded those with overt/occult gastrointestinal bleeding, with malnutrition and those received iron supplementation, etc., that some anemia patients related to nutritional deficiency or iron deficiency were eliminated from the cohort. Thus, the anemia prevalence in this study is relatively low.

We found no significant association between *H. pylori* infection and serum iron and ferritin levels. The question as to whether *H. pylori* infection plays a role in IDA has been widely studied. One updated systematic review and meta-analysis study has indicated an association between *H. pylori* infection and an increased likelihood of depleted iron storage [8]. On the contrary, some conflicting findings have been reported. A study in Australia of individuals aged ≥65 years found no significant association, except among female aspirin users, which supports a negative effect of *H. pylori* on iron status [18]. A recent large cohort retrospective study also reported that after adjusting for potential confounders, subjects with *H. pylori* infection had a higher prevalence of normocytic anaemia [27]. Patients with gastrointestinal bleeding or those received iron supplementation were excluded in this study, the level of iron deficiency could be underestimated. *H. pylori* infection could induce mixed deficiencies such as concomitant iron and vitamin B12 or folate deficiency, that would result in normocytic anemia. Owing to the normal iron and ferritin levels, as well as the normal erythroid-related parameters, we could exclude iron deficiency as a direct cause of normocytic and normochromic anemia in this study group. The absence of macrocytosis can help to infer that there might not be vitamin B12 deficiency in this cohort.

Apart from hematologic malignancies which accounted for 4% of anemia cases, some common causes of normocytic anemia encountered in the elderly included chronic inflammation, mixed nutrition deficiencies, chronic diseases and hemoglobinpathies [31]. Chronic inflammation is one of the most common causes of anemia in the elderly [33]. This type of anemia is typically normocytic and normochromic with mild to moderate reductions in hemoglobin level. Anemia of chronic inflammation is believed to be driven by immune responses [31, 34]. It is reported that *H. pylori* infection can cause systemic immune responses and chronic inflammation, that would subsequently induce the release of cytokines and inflammatory mediators such as interleukins 1 and 17, as well as tumor necrosis factor–α and interferon-γ [35]. The work-up and diagnosis of unexplained anemia in the elderly may include the measurement of inflammatory markers such as CRP and erythrocyte sedimentation rate, interleukin-6, and hepcidin levels [36]. It is reported that *H. pylori* infection may increase the level of serum CRP [37]. In this study, although all the subjects presented CRP levels within the normal range, we revealed a higher serum CRP level in the *H. pylori* positive group than that in the negative group. The possible attribution of *H. pylori* infection to changes in serum CRP level could be through a complex route of inflammation that has yet to be elucidated.

It is noticeable in this study that in the *H. pylori* positive subjects of higher CCI scores (>2), the prevalence of anemia was significantly higher than the negative ones with higher CCI scores (10.3% vs. 1.4%). This indicates that *H. pylori* infection could have a strong association with anemia in people with more comorbidities. No independent effect of CCI scores on anemia or hemoglobin level was found in our study, which was different from what the literature reported [38]. This may be because the average CCI score (mean = 1.93) was low in this cohort, which means that the subjects in this study are in relatively good health, their comorbidities are not severe enough to affect their anemia prevalence. These subjects are from well-developed areas in China, who are also at a social-economic level much better than average as evident by their ability to take part in a routine annual health checkup. This can also partially explain the findings in this study that the prevalence of *H. pylori* infection was 35.2%, which were lower than those cited by other studies conducted on the Chinese adult population [39]. The two similar CCI scores (1.85 vs. 1.97) in *H. pylori* positive and negative groups also helped to verify the effects of *H. pylori* infection, rather than comorbidities, on anemia in this special cohort.

Our study has several limitations. First, we used only ^13^C-UBT to determine current active *H. pylori* infection, which is believed to have better sensitivity and specificity than the serological assays [40]. ^13^C-UBT would present more false negative results in elderly population with more prevalence of atrophic gastritis [41], which may have biased the detection of *H. pylori* infection. It would be more meaningful if we have serological data and the patients could be classified into *H. pylori* negative, past *H. pylori* infection and current *H. pylori* infection subgroups. Second, we did not obtain the data on serum folate, vitamin B12 levels, which can potentially confound the causal relationship, although the absence of macrocytosis can help to address the issue. Besides, some residual confounders such as smoking and drinking are lacking, which may bias the analysis results. *H. pylori* eradication can increase the hemoglobin level in IDA patients [42], it would be meaningful if we can improve the normocytic and normochromic anemia after eradication in our cohort. This will be performed in our future study. Third, due to the nature of our institution, we could only study a relatively small sample, and limited to male subjects with a higher-than-average socio-economic status. Therefore, the results and conclusion are not representative of the population, even for the elderly, as a whole. Finally, we cannot confirm the actual causality between *H. pylori* infection and anemia as this is an observational study. Our hope is that other institutions will perform similar studies so that we can put together a more complete picture of how *H. pylori* infection affects the other clinical aspects of the elderly population.

## Conclusion

In summary, our data seem to suggest a positive association between active *H. pylori* infection, as determined by ^13^C-UBT, and anemia in an elderly asymptomatic male cohort, especially in those with more comorbidities. No difference was observed between ^13^C-UBT positive and negative subjects with respect to erythroid-related indices, serum iron or ferritin. More detailed studies are needed and could help to delineate the prevalence, and the mechanism of action that may lead to anemia after chronic *H. pylori* infection in the elderly.

## Appendix

**Table 5:** Summary of comorbidities measured in Charlson Comorbidity Index

	Number of cases (%)		
	*H. pylori* positive (*n* = 228)	*H. pylori* negative (*n* = 418)	Total (n = 646)
Myocardial infaction	5(2.2)	13(3.1)	18
Congestive heart failure	3(1.3)	6(1.4)	9
Peripheral vascular disease	90 (39.5)	137(32.8)	227
Cerebrovascular disease	40(17.5)	89(21.3)	129
Dementia	2(0.9)	1(0.2)	3
COPD	47(20.6)	57(13.6)	104
Connective tissue disease	1(0.4)	4(1.0)	5
Peptic ulcer disease	2(0.9)	2(0.5)	4
Diabetes mellitus	63(27.6)	117(28.0)	180
Moderate to severe chronic kidney disease	19(8.3)	45(10.8)	64
Hemiplegia	0	0	0
Leukemia	0	0	0
Malignant lymphoma	0	1(0.2)	1
Solid tumor^a^	22(9.6)	75(18.0)	97
Liver disease^b^	44(19.3)	85(20.3)	129
AIDS^c^	0	0	0
Total	338	632	970

^a^Excluding gastrointestinal malignant tumors

^b^Only 2 cases of hepatic cirrhosis were presented, with no hypersplenism

^c^Acquired immunodeficiency syndrome

**Table 6:** Data of 21 anemia patients

No.	CCI score	Tumor	age	DOB	H. pylori	Hemoglobin (g/L)	MCHC (g/L)	MCH (pg)	MCV (fl)	RBC (×10e12/L)	HCT (L/L)	Serum iron (μmol/L)	Serum ferritin (ng/mL)
1	4	0	85	1.2	0	116	335	33.4	99.7	3.47	0.346	16.3	86.4
2	0	0	73	23.9	1	114	328	28.4	86.8	4.01	0.348	11.3	206.908
3	3	0	75	4.9	1	118	336	29.3	87.1	4.03	0.351	15	76.73
4	3	0	94	4.8	1	118	311	29.4	94.5	4.01	0.379	18	193.5
5	2	0	74	4.1	1	109	344	31.4	91.4	3.47	0.317	18.5	44.12
6	2	0	72	12.8	1	112	327	32.8	100.3	3.41	0.342	33.9	779.3
7	1	0	87	0.5	0	109	321	31.2	97.4	3.49	0.34	9.1	411.4
8	1	0	69	4.3	1	111	327	30.4	92.9	3.65	0.339	15.5	47.08
9	0	0	81	0.6	0	115	323	31	96	3.71	0.356	18.9	98.31
10	2	0	68	0.1	0	118	326	29.6	90.7	3.99	0.362	13.2	89.63
11	1	0	73	1.7	0	109	312	27.6	88.4	3.95	0.349	7.6	22.93
12	6	0	93	31.8	1	115	325	31	95.4	3.71	0.354	8.3	141.9
13	4	0	76	22.3	1	101	334	32.9	98.4	3.07	0.302	16.3	71.28
14	3	0	91	38.3	1	119	324	30.7	94.8	3.87	0.367	15.7	90.86
15	3	0	82	4.9	1	108	325	28.6	87.8	3.78	0.332	13.5	206.908
16	3	0	89	3.5	0	102	324	33	101.9	3.09	0.315	10.3	399.9
17	2	0	73	11	1	117	346	32.1	92.6	3.65	0.338	11.8	140.3
18	1	0	90	5.5	1	118	341	33.3	97.7	3.54	0.346	21.3	170.7
19	1	0	70	1	0	110	322	31.8	98.8	3.46	0.342	18.2	57.82
20	1	0	76	0.1	0	117	367	34.8	94.9	3.36	0.319	16.2	146.5
21	0	0	70	0.5	0	111	339	30.2	88.9	3.68	0.327	12.9	350.5

Note: Tumor: the status of advanced malignant tumor, 0-negative; 1-positive; The status of H. pylori: 0-negative;1-positive; DOB: delta over baseline value of ^13^C-UBT

**Table 7:** Anemia prevalence (using hemoglobin 130 g/L as cutoff value) according to *H. pylori* status

*H. pylori* status	n (%)	OR	95%CI	*P* value
positive	31 (13.6)	1.27	0.78–2.07	0.332
negative	46 (11.0)	1.00 (Ref)		

**Table 8:** Effect of *H. pylori* status on erythroid-related index

Variables	Number of cases (%)		*P* value
	*H. pylori* positive	*H. pylori* negative	
MCHC			.582
< 320 g/L	9 (3.9)	20 (4.8)	
320–360 g/L	212 (93.0)	390 (93.3)	
> 360 g/L	7 (3.1)	8 (1.9)	
MCH			.987
< 27 pg	2 (0.9)	4 (1.0)	
27–34 pg	223 (97.8)	408 (97.6)	
> 34 pg	3 (1.3)	6 (1.4)	
MCV			.544
< 80 fl	0 (0.0)	2 (0.5)	
80–100 fl	224 (98.2)	407 (97.4)	
> 100 fl	4 (1.8)	9 (2.2)	
RBC			.372
< 3.5*10e12/L	4 (1.8)	7 (1.7)	
3.5–5.5*10e12/L	214 (93.9)	381 (91.1)	
> 5.5*10e12/L	10 (4.4)	30 (7.2)	
HCT			.058
< 0.35 L/L	21 (9.2)	20 (4.8)	
0.35–0.52 L/L	205 (89.9)	396 (94.7)	
> 0.52 L/L	2 (0.9)	2 (0.5)	
RDW			.391
< 14.5%	2 (0.9)	7 (1.7)	
≥ 14.5%	226 (99.1)	411 (98.3)	

## Abbreviations

^13^C-UBT: ^13^C-urea breath test
AIDS: Acquired immunodeficiency syndrome
CCI: Charlson comorbidity index
CIs: Confidence intervals
CRP: C-reactive protein
DOB: Delta over baseline value of ^13^C-UBT
*H. pylori*: *Helicobacter pylori*
HCT: Hematocrit
IDA: Iron deficiency anemia
MCH: Mean corpuscular hemoglobin
MCHC: Mean corpuscular hemoglobin concentration
MCV: Mean corpuscular volume
OR: Odds ratio
PPI: Proton pump inhibitor
RBC: Red blood cell
RDW: Red blood cells volume distribution width
